# Factors associated with knowledge and hypoglycemia experience among patients with diabetes mellitus in Ghana: A cross‐sectional study

**DOI:** 10.1002/puh2.130

**Published:** 2023-10-21

**Authors:** Samuel Asamoah Sakyi, Stephen Opoku, Ebenezer Senu, Emmanuel Ekow Korsah, Alfred Effah, Bright Takyi Baidoo, Eugene Ansah Arele, Emmanuel Frimpong, Emmanuel Naturinda, Kini Evans Kodzo, Anthony Amenuvor, Afia Agyapomaa Kwayie, Lydia Oppong Bannor, Ransford Osei Ampofo, Brefo Aaron Marfo, Raphael Osei Mensah‐Bonsu

**Affiliations:** ^1^ Department of Molecular Medicine School of Medicine and Dentistry Kwame Nkrumah University of Science and Technology Kumasi Ghana; ^2^ Division of Clinical Immunology and Rheumatology University of Alabama at Birmingham Birmingham UK; ^3^ Department of Medical Diagnostics Faculty of Allied Health Sciences College of Health Sciences Kwame Nkrumah University of Science and Technology Kumasi Ghana

**Keywords:** alcohol, diabetes mellitus, hypoglycemia, knowledge

## Abstract

**Background:**

Among persons with diabetes on treatment, hypoglycemia is the most common iatrogenic acute metabolic complication. Many factors influence hypoglycemia, highlighting the need for diabetic patients to recognize, and manage these potential factors in order to reduce the rate of hypoglycemia. In this study, we assessed the knowledge, experiences of hypoglycemia, and associated risk factors among Ghanaians with diabetes mellitus (DM).

**Methods:**

This cross‐sectional study included 444 clinically diagnosed DM patients from the Suntreso Government Hospital, who were on treatment for at least a year. A structured validated questionnaire was used to collect sociodemographic, lifestyle, and clinical data from the participants. Statistical analyses were performed using SPSS Version 20.0 and GraphPad Prism 8.0.

**Results:**

More than half (52.7%) of the study participants had poor knowledge of hypoglycemia. Moreover, 52.9% of diabetics had experienced hypoglycemia. Participants who were in the age groups of 55–64 and 65–80 years, being retired, being diagnosed with diabetes for 11–20 years and more than 20 years, taking in alcohol, and having adequate knowledge were independent determinants of experiencing hypoglycemia.

**Conclusion:**

Knowledge level of hypoglycemia among Ghanaian diabetics is low. Prompt recognition of risk factors of hypoglycemia and the careful monitoring and management of glycemic levels in high‐risk groups are important to lessen the prevalence of hypoglycemia in these populations.

## Graphical abstract

In persons with diabetes on treatment, hypoglycemia is the most common iatrogenic acute metabolic complication. This study found that knowledge level on hypoglycemia among Ghanaian diabetics is low, and hypoglycemia is influenced by age, occupation, duration of diabetes, and intake of alcohol as well as associated with hypoglycemia knowledge level.

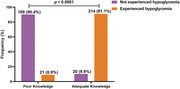

## INTRODUCTION

Diabetes mellitus (DM) is a global public health concern, characterized by gradual rise in its prevalence and mortality [1]. Globally, diabetes affects an estimated 537 million adults, with projections indicating a rise to 643 million by 2030 and 783 million by 2045 [2]. Majority of individuals with diabetes reside in lower and middle‐income countries, where a lack of awareness contributes to increase this health problem [2, 3]. In Africa, around 24 million adults have diabetes, resulting in approximately 416,000 reported deaths [2]. Trends in the prevalence of diabetes in Ghana do not differ significantly from those observed in other sub‐Saharan countries, with a rise in prevalence from 0.2% in 1958 [4] to an estimated prevalence of 6.46% in 2018 [5].

The high rates of morbidity and mortality observed in individuals with DM are mostly attributed to the occurrence of adverse complications. Diabetic ketoacidosis, hyperosmolar hyperglycemic state, gestational diabetes, and hypoglycemia are the most serious acute metabolic complications of the disease [6, 7, 8], although generally preventable.

Hypoglycemia is the most prevalent iatrogenic acute metabolic event among both type 1 and type 2 DM patients [9, 10], which affects patient safety and glycemic control during the therapeutic management of diabetes. It is defined as a condition where blood glucose levels drop below 70 mg/dL (3.9 mmol/L). Nevertheless, individuals may not experience any symptoms until their plasma glucose concentration falls below 55 mg/dL (3.1 mmol/L), putting the patients at risk of severe episodes [11, 12]. Symptoms and complications can vary from person to person and may change over time. These clinical manifestations are commonly categorized into adrenergic symptoms, such as shakiness, anxiety, nervousness, sweating, or pallor diaphoresis, hunger, and parentheses [13, 14] and neuroglycopenic symptoms, including ataxia, confusion, speaking problems, seizures, coma, and in most extreme cases, death [6, 13].

In Ghana, previous study has reported hypoglycemia as the second most frequent acute diabetic complication [7], whereas another has reported hypoglycemia as the most prevalent complication among diabetics [15]. This high prevalence of hypoglycemia and the evident poor awareness of hypoglycemia render diabetic patients prone to severe hypoglycemia and its related morbidity [16]. Hence, it is crucial to recognize the relationship between hypoglycemia and various factors, such as the patient's previous hypoglycemic events, knowledge of glycemic management, and the hypoglycemic event itself. Such insight can aid in reducing or even eradicating the possible adverse effects on individuals with DM undergoing therapy [17].

However, studies investigating the knowledge of hypoglycemia among diabetics in Ghana are lacking. Hence, this study assessed the knowledge of hypoglycemia, reported experiences of hypoglycemia, and associated risk factors among Ghanaians with DM.

## METHODS

### Study design and setting

This cross‐sectional study was conducted between January and September 2022 among patients with diabetes receiving treatment at the Diabetes Support Centre, Suntreso Government Hospital, Ghana. The Suntreso Government Hospital is in Kwadaso Municipality in the Ashanti Region of Ghana. The population of Kwadaso Municipality was 154,526, comprising 75,205 (48.7%) males and 79,321 (51.3%) females in 2021 [18]. The Suntreso Diabetes Support Centre was inaugurated in December 2018 with support from the Danish Embassy in Ghana.

### Study population

The Diabetes Support Centre caters for about 619 patients with diabetes. The study interviewed 444 patients with diabetes, aged 18 years and above, receiving various diabetes treatments for at least 12 months. Pregnant and breastfeeding mothers with diabetes were excluded. Patients with severe mental illnesses or malignant diseases and other issues resulting in an obstacle to completing the written questionnaire were also excluded.

### Data collection

A structured interview questionnaire was used to collect data from the participants. The questionnaire had five parts: sociodemographic profile, clinical and health profiles, lifestyle information, hypoglycemia symptoms, and knowledge on hypoglycemia. The study questionnaire was translated into the Akan language for easy comprehension among the diabetic patients. It was piloted among 10 patients who were excluded from the final study. It was back translated, and all inconsistencies and wrong wordings were corrected. The events of hypoglycemia were confirmed from patients’ folders and hospital registry.

### Scoring of knowledge on hypoglycemia

To assess the level of knowledge on hypoglycemia, a total of eight items structured questionnaire was used: the possibility of hypoglycemic episodes in the diabetic patients, precipitating factors, and common symptoms. All questions were based on previous validated questions [19] with modifications and the standards of medical care in diabetes by the American Diabetes Association [20]. A correct response was scored 1 and an incorrect response, 0. Participants with a score of 0–5 were classified as having poor knowledge, whereas a score of 6 or more was classified as having adequate knowledge.

### Data management and statistical analysis

The data were entered, cleaned, and coded using Microsoft Excel 2019. All statistical analyses were done using the Statistical Package for Social Sciences (SPSS) Version 26.0 and GraphPad Prism version 8.0 (GraphPad Software, www.graphpad.com). Categorical variables were presented as frequencies and percentages. Comparisons across sociodemographics, clinical and lifestyle characteristics, and hypoglycemia events were performed using a chi‐square. Logistic regression analysis was performed to screen for potential clinical and sociodemographic characteristics associated with knowledge of hypoglycemia and hypoglycemia experience. An adjusted logistic regression model was used to determine independent predictors of knowledge of hypoglycemia and hypoglycemia experience. The predictors were considered statistically significant if *p* < 0.05.

### Ethics consideration

Ethical approval was obtained from the Ethics Committee of the prospective hospitals and The Committee on Human Research, Publication and Ethics, School of Medical Sciences, Kwame Nkrumah University of Science and Technology (CHRPE/SMS/KNUST/CHRPE/AP/369/22) before the commencement of the study. Study protocols were duly explained to participants before data collection.

## RESULTS

A total of 444 participants were included in the study. The majority of the participants were females (77.5%) and resided in the urban areas (77.7%). Age group, marital status, number of children, level of education, and employment status were significantly associated with experiencing hypoglycemia (Table 1).

**TABLE 1:** Sociodemographic characteristics of patients with diabetes receiving treatment at the Diabetes Support Centre, Suntreso Government Hospital, Ghana, between January and September 2022.

Variables	Total (*n*, %)	Hypoglycemia (*n*, %)	*p* Value^a^
**Age groups (years)**			**<0.001**
25–44	55 (12.4)	8 (3.4)	
45–54	106 (23.9)	25 (10.6)	
55–64	143 (32.3)	87 (37.0)	
65–80	140 (31.5)	115 (48.9)	
**Gender**			0.497
Male	100 (22.5)	56 (23.8)	
Female	344 (77.5)	179 (76.2)	
**Marital status**			**<0.001**
Married	250 (56.3)	116 (49.4)	
Widowed	92 (20.7)	59 (25.1)	
Divorced	74 (16.7)	54 (23.0)	
Single	28 (6.3)	6 (2.6)	
**Number of children**			**<0.001**
0–2	104 (23.4)	28 (11.9)	
2–5	289 (65.1)	174 (74.0)	
6–9	51 (11.5)	33 9 (14.0)	
**Residence**			0.211
Urban	345 (77.7)	177 (75.3)	
Rural	99 (22.3)	58 (24.7)	
**Level of education**			**<0.001**
No formal education	187 (42.1)	132 (56.2)	
Primary	27 (6.1)	13 (5.5)	
Secondary	178 (40.1)	67 (28.5)	
Tertiary	52 (11.7)	23 (9.8)	
**Ethnicity**			0.972
Akan	363 (81.8)	193 (82.1)	
Northerner	39 (8.8)	20 (8.5)	
Ewe	42 (9.5)	22 (9.4)	
**Employment**			**<0.001**
Employed	69 (15.5)	20 (8.5)	
Self‐employed	252 (56.8)	125 (53.2)	
Unemployed	107 (24.1)	75 (31.9)	
Retired	16 (3.6)	15 (6.4)	

^a^ Chi‐square or Fisher's test. *p*‐Values < 0.05 and bolded means statistically significant.

The majority of the patients almost never checked home test their glucose levels (77.5%). Most of the study participants were on metformin‐based regimen (96.2%), had type 2 DM (72.7%), never exercise (72.7%), and were non‐alcoholics (86.0%). The duration of diabetes, frequency of glucose checks, exercise level, and alcohol intake were significantly associated with experiencing hypoglycemia (Table 2).

**TABLE 2:** Clinical and lifestyle characteristics of patients with diabetes receiving treatment at the Diabetes Support Centre, Suntreso Government Hospital, Ghana, between January and September 2022.

Variables	Total (*n*, %)	Hypoglycemia (*n*, %)	*p* Value^a^
**Family history of diabetes**	64 (14.4)	31 (13.2)	0.499
**Duration of diabetes (years)**			**<0.001**
1–10	285 (64.2)	114 (48.5)	
11–20	108 (24.3)	76 (32.3)	
≥21	51 (11.5)	45 (19.1)	
**Frequency of glucose checks**			**0.019**
Daily	38 (8.6)	15 (6.4)	
Weekly	40 (9.0)	17 (7.2)	
Occasionally	22 (5.0)	17 (7.2)	
Almost never	344 (77.5)	186 (79.1)	
**Current medicine**			0.080
Metformin	427 (96.2)	230 (97.9)	
Insulin	17 (3.8)	5 (2.1)	
**Type of diabetes**			0.080
Type 1	17 (3.8)	5 (2.1)	
Type 2	427 (96.2)	230 (97.9)	
**Exercise level**			**<0.001**
Daily	38 (8.6)	12 (5.1)	
Weekly	35 (7.9)	17 (7.2)	
Occasionally	48 (10.8)	16 (6.8)	
Never	323 (72.7)	190 (80.9)	
**Alcohol intake**	62 (14.0)	24 (10.2)	**0.019**

^a^ Chi‐square or Fisher's test. *p*‐Values < 0.05 and bolded means statistically significant.

### Knowledge of hypoglycemia among T2DM patients

Responses to study participants to the standard hypoglycemia knowledge questionnaire are shown in Table 3.

**TABLE 3:** Knowledge of hypoglycemia and status among T2DM patients receiving treatment at the Diabetes Support Centre, Suntreso Government Hospital, Ghana, between January and September 2022.

Knowledge domains	Correct response *n* (%)	Incorrect response *n* (%)
Can hypoglycemia occur in people with diabetes?	338 (76.1)	106 (23.9)
Can hypoglycemia episodes prove to be dangerous?	326 (73.4)	118 (26.6)
Can hypoglycemia be precipitated by skipping meals or excessive exercises?	262 (59.0)	182 (41.0)
Is morning headache one of the symptoms of hypoglycemia?	179 (40.3)	265 (59.7)
Is shakiness one of the symptoms of hypoglycemia?	327 (73.6)	117 (26.4)
Is intense hunger one of the symptoms of hypoglycemia?	162 (36.5)	282 (63.5)
Is passing out one of the symptoms of hypoglycemia?	228 (51.4)	216 (48.6)
Is body weakness one of the symptoms of hypoglycemia?	261 (58.8)	183 (41.2)

**Overall knowledge level**	**Frequency (*n*)**	**Percentage (%)**
Poor knowledge	234	52.7
Adequate knowledge	210	47.3

**Hypoglycemic status**	**Frequency (*n*)**	**Percentage (%)**
Not experienced hypoglycemia	209	47.1
Experienced hypoglycemia	235	52.9

A total knowledge score was computed, and a near majority of patients had poor knowledge of hypoglycemia (234, 52.7%), whereas 210 (42.7%) had adequate knowledge of hypoglycemia (Table 3).

### Proportion of hypoglycemia experience among DM patients

Of the proportion of DM patients who had experienced hypoglycemia, more than half of the study participants had experienced hypoglycemia (52.9%), whereas 47.1% had not experienced hypoglycemia (Table 3).

### Proportion of knowledge stratified by hypoglycemia experiences

**FIGURE 1:**
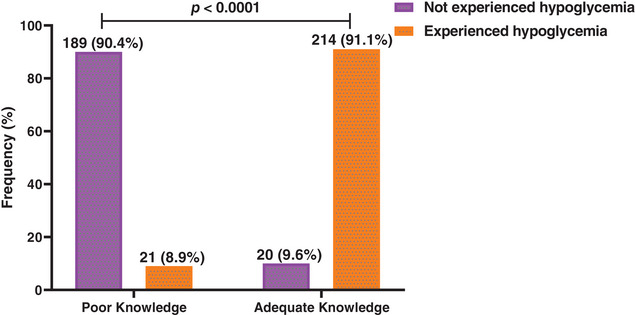
Proportion of knowledge level stratified by hypoglycemia experiences among diabetes mellitus patients receiving treatment at the Diabetes Support Centre, Suntreso Government Hospital, Ghana, between January and September 2022.

This study observed that the majority of diabetic patients with adequate knowledge on hypoglycemia had experienced hypoglycemia, whereas majority with poor knowledge had not experienced hypoglycemia. Knowledge of hypoglycemia was therefore significantly associated with experiencing hypoglycemia among study participants (Figure 1).

### Predictors of poor hypoglycemia knowledge

In the multivariate logistic regression analysis, being in the age groups of 55–64 [adjusted OR = 0.29, 95% CI: (0.12–0.73), *p* = 0.008] and 65–80 [adjusted OR = 0.10, 95% CI: (0.36–0.27), *p* < 0.001] years was independently associated with a decrease in poor knowledge of hypoglycemia. Moreover, DM patients who experienced hypoglycemia [adjusted OR = 2.92, 95% CI: (1.03–4.31), *p* < 0.001] were at 3‐odds of having poor knowledge of hypoglycemia (Table 4).

**TABLE 4:** Predictors of poor knowledge level on hypoglycemia among patients with diabetes receiving treatment at the Diabetes Support Centre, Suntreso Government Hospital, Ghana, between January and September 2022.

	Unadjusted analysis^a^		Adjusted analysis^b^	
Variables	OR (95% CI)	*p* Value	Adjusted OR (95% CI)	*p* Value
**Age groups (years)**				
25–44	1.00		1.00	
45–54	0.95 (0.46–1.97)	0.840	0.91 (0.38–2.18)	0.840
55–64	0.27 (0.14–0.54)	<0.001	0.29 (0.12–0.73)	**0.008**
65–80	0.12 (0.060–0.24)	<0.001	0.10 (0.36–0.27)	**<0.001**
**Marital status**				
Married	0.53 (0.23–1.22)	0.134	1.82 (0.63–5.33)	0.271
Widowed	0.33 (0.14–0.82)	0.016	2.82 (0.84–9.43)	0.092
Divorced	0.19 (0.07–0.48)	<0.001	1.04 (0.31–3.52)	0.950
Single	1.00		1.00	
**Number of children**				
0–2	1.00		1.00	
2–5	0.36 (0.26–0.58)	<0.001	0.72 (0.39–1.35)	0.308
6–9	0.36 (0.18–0.71	0.003	0.81 (0.35–1.90)	0.629
**Level of education**				
No formal education	0.54 (0.29–0.99)	0.050		
Primary	1.57 (0.613–4.03)	0.347		
Secondary	1.63 (0.87–3.03)	0.124		
Tertiary	1.00			
**Employment**				
Employed	1.00		1.00	
Self‐employed	0.49 (0.28–0.86)	0.012	0.79 (0.43–1.45)	0.443
Unemployed	0.31 (0.16–0.58)	<0.001	1.03 (0.47–2.26)	0.947
Retired	0.24 (0.08–0.78)	0.017	0.81 (0.23–2.84)	0.736
**Duration of diabetes (years)**				
1–10	1.00		1.00	–
11–20	0.34 (0.22–0.55)	<0.001	1.41 (0.45–4.37	0.554
≥21	0.15 (0.07–0.33)	<0.001	1.06 (0.32–3.54)	0.931
**Exercise level**				
Daily	1.00		1.00	
Weekly	0.44 (0.17–1.13)	0.141	0.47 (0.10–2.31)	0.353
Occasionally	0.73 (0.30–1.76)	0.863	0.49 (0.12–2.11)	0.341
Never	0.40 (0.20–0.82)	0.012	1.11 (0.33–3.78)	0.869
**Hypoglycemia experience**	2.98 (1.10–3.95)	<0.001	2.92 (1.03–4.31)	**<0.001**

Abbreviations: Inf, infinity; OR: odds ratio.

^a^ Compared using univariate logistic regression.

^b^ Compared using multivariate logistic regression. *p*‐Values < 0.05 and bolded means statistically significant.

### Predictors of hypoglycemia

Table 5 shows the odds of association among sociodemographic, lifestyle, and clinical factors with experiencing hypoglycemia. After adjusting for possible confounders In the multivariate logistic regression model, being in the age groups of 55–64 [adjusted OR = 8.30, 95% CI: (2.66–25.84), *p* < 0.001] and 65–80 years [adjusted OR = 25.93, 95% CI: (7.45–90.22), *p* < 0.001], being retired [adjusted OR = 16.46, 95% CI: (1.80–153.97), *p* = 0.013], being diagnosed of diabetes for 11–20 years [adjusted OR = 2.79, 95% CI: (1.17–6.66), *p* = 0.021] and more than 20 years [adjusted OR = 6.53, 95% CI: (1.80–23.73), *p* = 0.004], taking in alcohol [adjusted OR = 0.33, 95% CI: (0.13–0.87), *p* = 0.025] and having adequate knowledge of hypoglycemia [adjusted OR = 114.88, 95% CI: (54.04–224.20), *p* < 0.001] were independent determinants of experiencing hypoglycemia (Table 5).

**TABLE 5:** Predictors of experiences of hypoglycemia among patients with diabetes receiving treatment at the Diabetes Support Centre, Suntreso Government Hospital, Ghana, between January and September 2022.

	Unadjusted analysis^a^		Adjusted analysis^b^	
Variables	OR (95% CI)	*p* Value	Adjusted OR (95%) CI	*p* Value
**Age groups (years)**				
25–44	1.00		1.00	
45–54	1.81 (0.76–4.34)	0.182	2.05 (0.69–6.11)	0.198
55–64	9.13 (4.01–20.75)	<0.001	8.30 (2.66–25.84)	**<0.001**
65–80	27.03 (11.37–64.21)	<0.001	25.93 (7.45–90.23)	**<0.001**
**Marital status**				
Married	3.17 (1.25–8.10)	0.016	0.64 (0.340–1.20)	0.165
Widowed	6.56 (2.42–17.79)	<0.001	1.62 (0.82–3.19)	0.167
Divorced	9.90 (3.51–27.96)	<0.001	2.55 (0.71–9.25)	0.153
Single	1.00		1.00	–
**Number of children**				
0–2	1.00		1.00	
2–5	4.11 (2.51–6.73)	<0.001	1.45 (0.73–2.91)	0.291
6–9	4.98 (2.43–10.23)	<0.001	1.38 (0.53–3.56)	0.507
**Level of education**				
No formal education	3.03 (1.61–5.69)	0.001	0.62 (0.22–1.79)	0.378
Primary	1.17 (0.46–2.98)	0.740	0.50 (0.14–1.82)	0.290
Secondary	0.76 (0.41–1.42)	0.392	0.38 (0.15–1.01	0.051
Tertiary	1.00		1.00	
**Employment**				
Employed	1.00		1.00	
Self‐employed	2.41 (1.36–4.29)	0.003	1.88 (0.79–4.50)	0.155
Unemployed	5.74 (2.95–11.16)	<0.001	1.94 (0.70–5.37)	0.203
Retired	36.75 (4.55–297.12)	<0.001	16.46 (1.80–153.97)	**0.013**
**Duration of diabetes (years)**				
1–10	1.00		1.00	
11–20	3.56 (2.21–5.74)	<0.001	2.79 (1.17–6.66)	**0.021**
≥21	11.25 (4.65–27.24)	<0.001	6.53 (1.80–23.73)	**0.004**
**Frequency of glucose checks**				
Daily	1.00	–	1.00	–
Weekly	1.13 (0.46–2.30)	0.786	0.69 (0.14–3.27)	0.637
Occasionally	5.2 (1.59–17.15)	0.007	2.79 (0.40–19.29)	0.299
Almost never	1.81 (0.91–3.58)	0.091	0.93 (0.29–3.31)	0.911
**Exercise level**				
Daily	1.00		1.00	
Weekly	2.05 (0.79–5.30)	0.141	1.39 (0.28–6.91)	0.685
Occasionally	1.08 (0.44–2.69)	0.863	0.68 (0.15–3.06)	0.616
Never	3.10 (1.51–6.35)	0.002	2.29 (0.69–7.56)	0.174
**Alcohol intake**	0.51 (030–0.89)	0.017	0.33 (0.13–0.87)	**0.025**
**Knowledge of hypoglycemia**				
Adequate	96.30 (50.63–183.16)	<0.001	114.88 (54.04–244.20)	**<0.001**
Poor	1.00		1.00	–

Abbreviation: OR, odds ratio.

^a^ Compared using univariate logistic regression.

^b^ Compared using multivariate logistic regression. *p*‐Values < 0.05 and bolded means statistically significant.

## DISCUSSION

In individuals receiving treatment for diabetes, hypoglycemia remains one of the most prevalent acute metabolic complications. It can lead to significant and potentially life‐threatening complications, including heart arrhythmias, cognitive impairment, and cerebral ischemia. Individuals with inadequate knowledge of hypoglycemia are vulnerable to experiencing severe hypoglycemia and its related morbidity.

This current study examined the knowledge regarding hypoglycemia and its association with experiencing hypoglycemia among diabetic patients at the Diabetes Support Centre, Suntreso Government Hospital. Additionally, we identified the sociodemographic and clinical factors associated with hypoglycemia knowledge and hypoglycemia experience.

In this study, although the majority knew hypoglycemia could occur in diabetic patient, 23.9% had no idea of this life‐threatening event. This is comparable to a study by Cho et al. in tertiary referral centers in Korea (22.1%) [21]. Most of the subjects agreed that hypoglycemic episodes can prove to be dangerous (73.4%) and can be precipitated by meals or excessive exercise (59.0%). Majority of patients recognized that shakiness (73.6%), passing out, and weakness were symptoms of hypoglycemia. However, less than half of the participant failed to recognize that morning headache (40.3%) and intense hunger (36.5%) are symptoms of hypoglycemia. Similar findings have been reported in several studies, which found poor knowledge of one or both of these common symptoms of hypoglycemia among DM patients [22, 23, 24]. Overall, 52.7% had poor knowledge of hypoglycemia. This finding was greater than reports by Shriraam et al. among DM patients in a tertiary hospital in India (33.9%) [22]. Adequate knowledge about hypoglycemia is essential for recognizing and preventing both hypoglycemia and its severe complication. Hypoglycemia education by health professionals during visits of DM patients is thus required in the Ghanaian settings; a habit that is mostly avoided in the management of diabetic patients in our setting.

In this present study, more than half of the study participants had experienced hypoglycemia (52.9%). This finding was higher than reports in South Africa (43.4%) [25]. However, it was lower than what was reported in Poland (62.0%) [26] and Korea (62.1%) [21]. These observed differences may be due to variations in sample size or ethnic background. Differences in the characterization of hypoglycemia may also account for this observed variation.

We also assessed the independent predictors of poor hypoglycemia knowledge among diabetics. We observed that older individuals with diabetes were less likely to have poor knowledge of hypoglycemia. This finding may be attributed to the fact that older adults have lived with diabetes for a longer time, giving them more experience and exposure to hypoglycemia. This means they have learned from their own experiences and healthcare providers about the occurrence, precipitating factors, and common symptoms of hypoglycemia.

We also found evidence for relationship between experiencing hypoglycemia and multiple sociodemographic characteristics among DM patients, including older age groups, long duration of DM, retired personnel, alcohol intake, and adequate hypoglycemia knowledge. Being aged 55 and above was independently associated with higher odds of experiencing hypoglycemia. This finding is consistent with those observed by previous studies [27, 28]. This finding highlights the importance of increased awareness and vigilance in the management of diabetes in this population. Several factors may contribute to the increased risk of hypoglycemia in older adults. Changes in metabolism, such as decreased insulin sensitivity and impaired counter‐regulatory hormones’ responses, may contribute to a higher risk of hypoglycemia in this population [29, 30]. Moreover, most adults present with multiple medical conditions, including hypertension, renal disease, and cognitive disorders, increasing their vulnerability to hypoglycemia. Drug interaction from the intake of multiple medications increases the risk of drug interactions and eventually contributing to hypoglycemia in these groups [28, 31]. In addition, older adults may be prone to changes in appetite and dietary habits, which can affect blood sugar levels. Moreover, DM patients who have had the disease for more than 21 years were independently associated with approximately sevenfold of having hypoglycemia. This is in agreement with reports from Zammitt et al. [27]. Long‐term diabetes can lead to changes in the body's sensitivity to insulin, making it more challenging to regulate blood sugar levels.

Retired personnel with DM are at a higher chance of experiencing hypoglycemia. These populations are also likely to have changes in their health status; the presence of comorbidities and changes in medication regimen, increasing the risk of hypoglycemia in this population. Further research is needed to investigate the underlying mechanisms and identify effective strategies for managing hypoglycemia risk in retired diabetic patients.

Surprisingly, alcohol intake was associated with a reduced risk of hypoglycemia. Other factors may play a role, such as the type of alcohol consumed, the timing of alcohol consumption, and overall health and lifestyle habits may also be involved [20, 26, 28]. Further studies assessing all these variables are needed to fully understand the effects of alcohol consumption on blood sugar control in individuals with diabetes.

The main finding of this study is that having adequate knowledge of hypoglycemia was associated with greater odds of experiencing hypoglycemia. This finding is consistent with a previous study, which found a low hypoglycemic knowledge score was more common among diabetic patients who do not experience hypoglycemic [17]. This emphasizes the significant role of knowledge in managing the risks associated with diabetes among patients.

The novelty of our study in Ghana provides important findings to guide healthcare personnel and policymakers in the management of Ghanaian DM patients. Nonetheless, there are a few limitations. Data were collected using questionnaires, the accuracy of which may be influenced by a recall bias. Recognizing the limitations of relying solely on participants’ recall, we incorporated cross‐referencing patients’ folders, providing clear instructions and using a validated questionnaire. The study was also conducted in a single health center. Hence, we acknowledge the need for further research that includes larger sample sizes and a more diverse range of healthcare settings to enhance the generalizability of the findings. Moreover, this is a cross‐sectional study; hence, causal relationship could not be established.

## CONCLUSION

Knowledge level of hypoglycemia among Ghanaian diabetics is low. The propensity to experience hypoglycemia is influenced by their age, occupation, duration of diabetes, and intake of alcohol. Hence, prompt recognition of these risk factors and closely monitoring high‐risk groups is crucial. Healthcare education at primary hospitals and technology advancement like continuous glucose monitors are vital for optimal diabetes management.

## AUTHOR CONTRIBUTIONS

*Conceptualization; investigation; methodology; supervision; writing—original draft; writing—review and editing*: Samuel Asamoah Sakyi. *Conceptualization; data curation; investigation; methodology; writing—original draft; writing—review and editing*: Stephen Opoku, Alfred Effah, Bright Takyi Baidoo, Kini Evans Kodzo, Anthony Amenuvor, Afia Agyapomaa Kwayie, Lydia Oppong Bannor, Ransford Osei Ampofo, Brefo Aaron Marfo, Raphael Osei Mensah‐Bonsu, and Emmanuel Naturinda. C*onceptualization; data curation; formal analysis; investigation; methodology; software; writing—original draft; writing—review and editing*: Ebenezer Senu. *Conceptualization; data curation; formal analysis; investigation; methodology; writing—original draft; writing—review and editing*: Emmanuel Ekow Korsah. *Conceptualization; investigation; methodology; writing—original draft; writing—review and editing*: Eugene Ansah Arele and Emmanuel Frimpong.

## CONFLICT OF INTEREST STATEMENT

Authors declare that no conflicts of interest exist.

## FUNDING INFORMATION

This study did not receive funding from private, government, or non‐for‐profit organizations.

## Data Availability

All data generated or analyzed during this study are included in this article and can be requested from the corresponding author.
